# Anterior cruciate ligament femoral-tunnel drilling through an anteromedial portal: 3-dimensional plane drilling angle affects tunnel length relative to notchplasty

**DOI:** 10.1186/s43019-021-00092-5

**Published:** 2021-04-14

**Authors:** Dong-Kyu Moon, Ho-Seung Jo, Dong-Yeong Lee, Dong-Geun Kang, Hee-Chan Won, Min-Seok Seo, Sun-Chul Hwang

**Affiliations:** 1 Department of Orthopaedic Surgery and Institute of Health Science, Gyeongsang National University School of Medicine, Gyeongsang National University Hospital, Jinju, Republic of Korea; 2 Department of Orthopaedic Surgery, Barun Hospital, Jinju, Republic of Korea; 3 Department of Orthopaedic Surgery, Gyeongsang National University Changwon Hospital, Changwon, Republic of Korea

**Keywords:** ACL, Femoral tunnel, Drilling angle, Notchplasty, Computer simulation

## Abstract

**Background:**

Notchplasty is a surgical technique often performed during anterior cruciate ligament reconstruction (ACLR) with widening of the intercondylar notch of the lateral distal femur to avoid graft impingement. The purpose of this study was to correlate femoral-tunnel length with 3-dimensional (3D) drilling angle through the anteromedial (AM) portal with and without notchplasty.

**Materials and methods:**

Computer data were collected from an anatomical study using 16 cadaveric knees. The anterior cruciate ligament (ACL) femoral insertion was dissected and outlined for gross anatomical observation. The dissected cadaveric knees were scanned by computed tomography (CT). Three-dimensional measurements were calculated using software (Geomagic, Inc., Research Triangle Park, NC, USA) and included the center of the ACL footprint and the size of the ACL femoral footprint. The femoral-tunnel aperture centers were measured in the anatomical posterior-to-anterior and proximal-to-distal directions using Bernard’s quadrant method. The ACL tunnel was created 3-demensionally in the anatomical center of femoral foot print of ACL using software (SolidWorks®, Corp., Waltham, MA, USA). The 8-mm cylinder shaped ACL tunnel was rested upon the anatomical center of the ACL footprint and placed in three different positions: the coronal plane, the sagittal plane, and the axial plane. Finally, the effect of notchplasty on the femoral-tunnel length and center of the ACL footprint were measured. All the above-mentioned studies performed ACLR using the AM portal.

**Results:**

The length of the femoral tunnels produced using the low coronal and high axial angles with 5-mm notchplasty became significantly shorter as the femoral starting position became more horizontal. The result was 30.38 ± 2.11 mm on average at 20° in the coronal plane/70° in the axial plane/45° in the sagittal plane and 31.26 ± 2.08 mm at 30° in the coronal plane/60° in the axial plane/45° in the sagittal plane, respectively, comparing the standard technique of 45° in the coronal/45° in the axial/45° in the sagittal plane of 32.98 ± 3.04 mm (*P* < 0.001). The tunnels made using the high coronal and low axial angles with notchplasty became longer than those made using the standard technique: 40.31 ± 3.36 mm at 60° in the coronal plane/30° in the axial plane/45° in the sagittal plane and 50.46 ± 3.13 mm at 75° in the coronal plane/15° in the axial plane/45° in the sagittal plane (*P* < 0.001).

**Conclusions:**

Our results show that excessive notchplasty causes the femoral tunnel to be located in the non-anatomical center of the ACL footprint and reduces the femoral-tunnel length. Therefore, care should be taken to avoid excessive notchplasty when performing this operation.

## Background

The importance of correct positioning in the sagittal plane in anterior cruciate ligament (ACL) reconstruction (ACLR) was recognized many years ago and incorrect positioning of the femoral tunnel yields poor clinical results [1, 2]. However, the importance of correct positioning of an ACL graft in the coronal plane has been underestimated. In the last few years, many authors have demonstrated the biomechanical advantages of recreating the obliquity of the ACL graft (like the native ACL) in the coronal plane [3–5].

Recent studies have shown that drilling of the femoral tunnel at the anatomical footprint site of the ACL results in a more oblique angle and a shorter femoral tunnel [6, 7]. According to many surgeons, correct placement of the femoral tunnel can be achieved using the transtibial (TT) technique [3, 8]. However, as has been demonstrated by Arnold et al. [9] TT femoral-tunnel drilling does not reach the anatomical site of the ACL insertion at the 10 o’clock position. Usually with the TT technique, a position corresponding to between the 11 and 12 o’clock positions could be reached, and the graft is then placed in a relatively vertical position. To overcome these problems, many authors recommend the anteromedial (AM) technique [10, 11]. Moreover, the AM portal technique provides more anatomical graft placement than the TT technique in ACLR. This study obtained computer data values using the AM portal, which has many advantages.

Notchplasty can be used during ACLR to improve visualization of the posterior wall, allow for easier passage of the graft, and to prevent impingement of the graft [12–14]. However, important bony landmarks for anatomical placement of the graft may be lost by using notchplasty, and incorrect tunnel placement may occur, which may negatively affect the biomechanics of the reconstructed ACL [15, 16]. Moreover, it is controversial regarding the location of where notchplasty should be performed and the amount of bone removed. In view of the current trend towards a more oblique femoral tunnel in anatomical ACLR [17]. Therefore, shortening and disorientation of the femoral tunnel after notchplasty might be problematic for contemporary ACLR. The purpose of this study was to correlate femoral-tunnel length, angle, and tunnel dimension with 3-dimensional (3D) drilling angle through the accessory AM portal with and without notchplasty. Our hypothesis was that there might be differences in tunnel length and change in the center of the ACL footprint according to different 3D entrance angles with and without notchplasty, which might affect the knee’s biomechanics and clinical outcome.

## Materials and methods

### 3D modeling of cadaveric knees

**Fig. 1:**
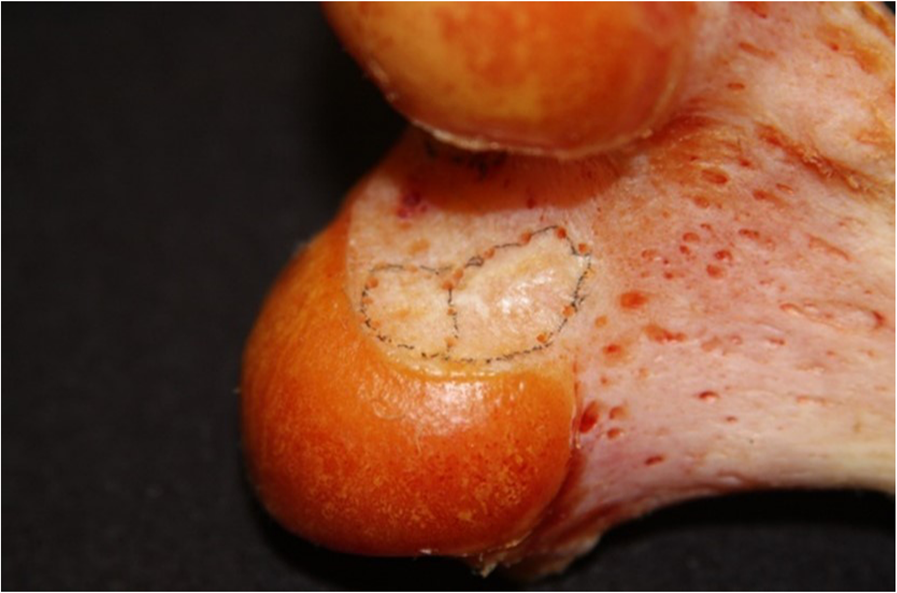
Main photograph: anterior cruciate ligament (ACL) insertion outlined on a femur

**Fig. 2:**
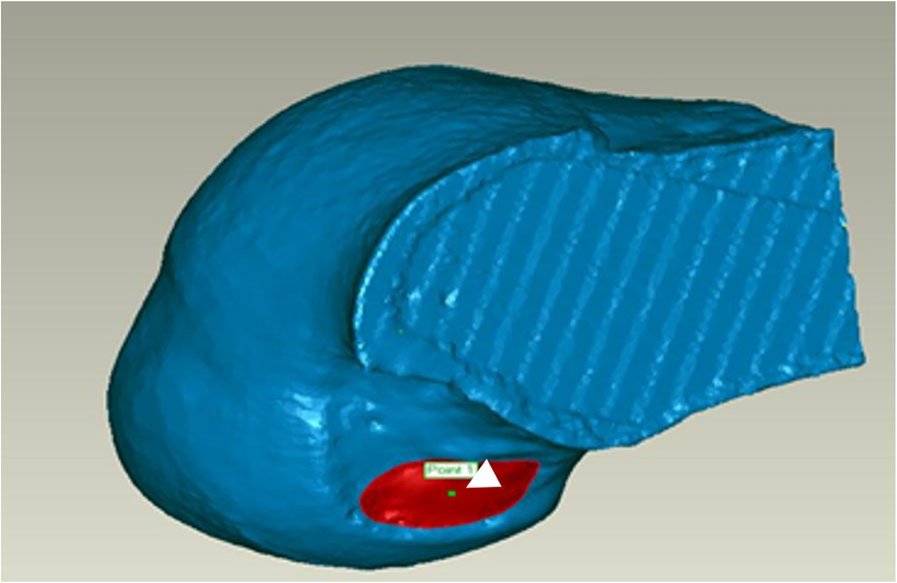
Three-dimensional (3D) measurements of the anterior cruciate ligament (ACL) footprint were calculated using software (Geomagic®, Inc., Research Triangle Park, NC, USA). The arrow head indicates the center of the ACL footprint

**Fig. 3:**
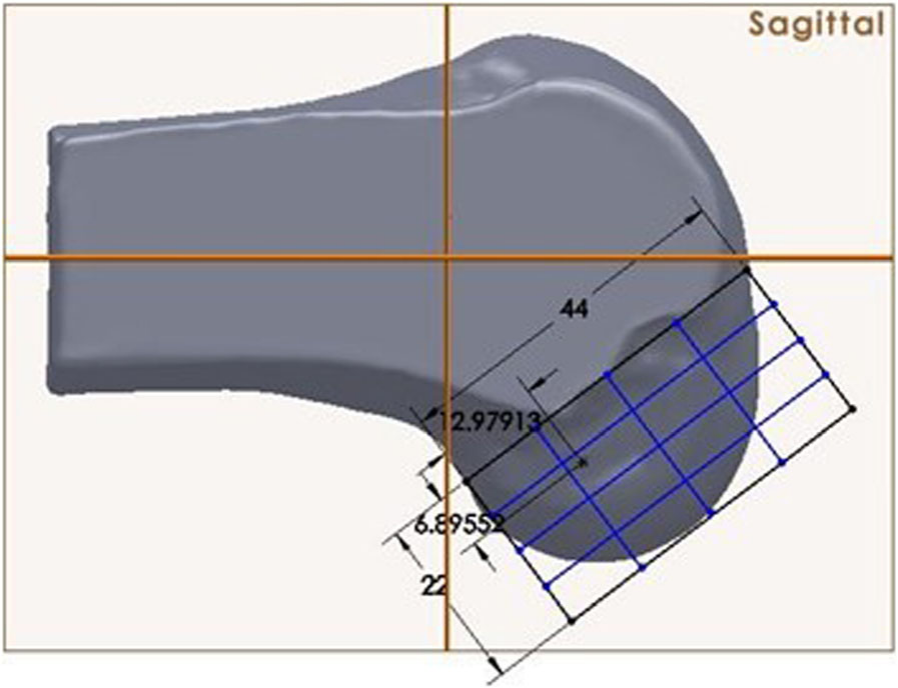
Femoral-tunnel aperture center measured in posterior-to-anterior and proximal-to-distal directions using Bernard’s quadrant method. Distance “*t*” is defined the total sagittal diameter of the lateral femoral condyle measured along Blumensaat’s line. It was limited by the intersections between this line and the ventral and dorsal borders of the femoral condyle. Also, distance “*h*” is defined as the height of the intercondylar space measured as the distance between Blumensaat’s line and a tangent to the distal subchondral bone contour of the condyle parallel to Blumensaat’s line. Software (SolidWorks®, SolidWorks® Corp., Waltham, MA, USA) is used to create a femoral tunnel at the anatomical center of the ACL and 3-dimensional planes (coronal, transverse, axial)

This was a pilot, experimental, cadaveric study. Cadavers with severe arthritis (Kellgren-Lawrence grade 3 or high as evaluated on computed tomography (CT) imaging) and knees that had undergone any previous surgery were excluded on this study. The anatomical structure of the femoral tunnel was analyzed using 16 cadaveric knees (12 male, 4 female). The mean subject age at the time of death was 65.4 ± 6.2 years (range 56–73 years). Each of the 16 cadavers underwent CT scanning, and a 3D stereoscopic model was obtained. All measurements were assumed when using the AM portal. The ACL femoral insertion was dissected and outlined for gross anatomical observation. The femoral ACL footprint outline was marked using a 1.5-mm drill bit (Fig. 1). Following the CT scan, a 3D model of each knee was reconstructed using MIMICS® software (Mimics 12.3, Materialise, Leuven, Belgium) to verify the positions and to virtually remove the medial femoral condyle to better visualize the lateral femoral condyle and ACL footprint. Three-dimensional measurements of the bony structure of the knee joint including the center of the ACL footprint and the size of the ACL femoral footprint were calculated using Geomagic® software (Research Triangle Park, NC, USA, see Fig. 2). The center of the ellipse-shaped anatomical ACL femoral footprint was automatically calculated by the computer program, which is based on CT images of the perimeter femoral ACL footprint marked previously. The femoral aperture centers were measured in the anatomical posterior-to-anterior and proximal-to-distal directions using Bernard’s quadrant method relative to the femoral notch (Fig. 3) [18].

### Creation of tunnel and notchplasty and structural measurement

Using SolidWorks® software (SolidWorks®, Corp., Waltham, MA, USA), ACL tunnels were created in the anatomical center of the ACL in the form of a diameter 8mm cylinder on the 3D plane. The authors set the angle based on the following criteria in order to check the effect of the angle at which the ACL tunnel was formed. The authors have selected 0° relative to an imaginary line connecting the distal margins of the femoral condyle. In addition, we selected the angle of insertion in the coronal plane of the tunnel of this baseline as the coronal angle. We also set a virtual line connecting the posterior margin of the lateral and medial femoral condyles to a standardized 0°. The axial angle was determined to be the angle of insertion in the axial plane of the tunnel at the baseline. We then set the plane perpendicular to the other two planes as the sagittal plane, the axis of the femoral shaft as 0°, and the insertion angle of the tunnel of this baseline as the sagittal angle. The 3D entrance angle was settled with five different angles randomly by using 16 cadaveric knees, which underwent anatomical, single-bundle ACLR, and the averaging angle at each plane under the computer simulation. The cylinder was rested upon the anatomical center of the ACL footprint and placed in five different position: at 20^o^, 30^o^, 45^o,^ 60^o^, and 75^o^ in the coronal plane, at 20^o^, 30^o^, 45^o,^ 60^o^, and 75^o^ in the axial plane and at 45^o^ in the sagittal plane.

**Fig. 4:**
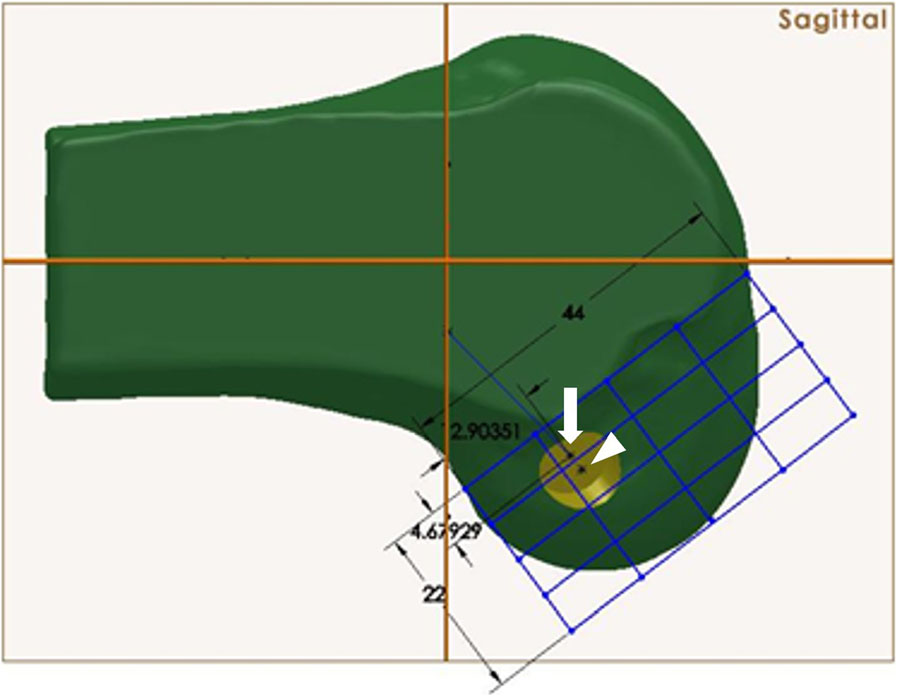
Notchplasty was created using software (SolidWorks®, SolidWorks®, Corp., Waltham, MA, USA). The arrow head indicates the native center of the anterior cruciate ligament (ACL) footprint. The arrow indicates the center of the ACL footprint that has changed after notchplasty

**Fig. 5:**
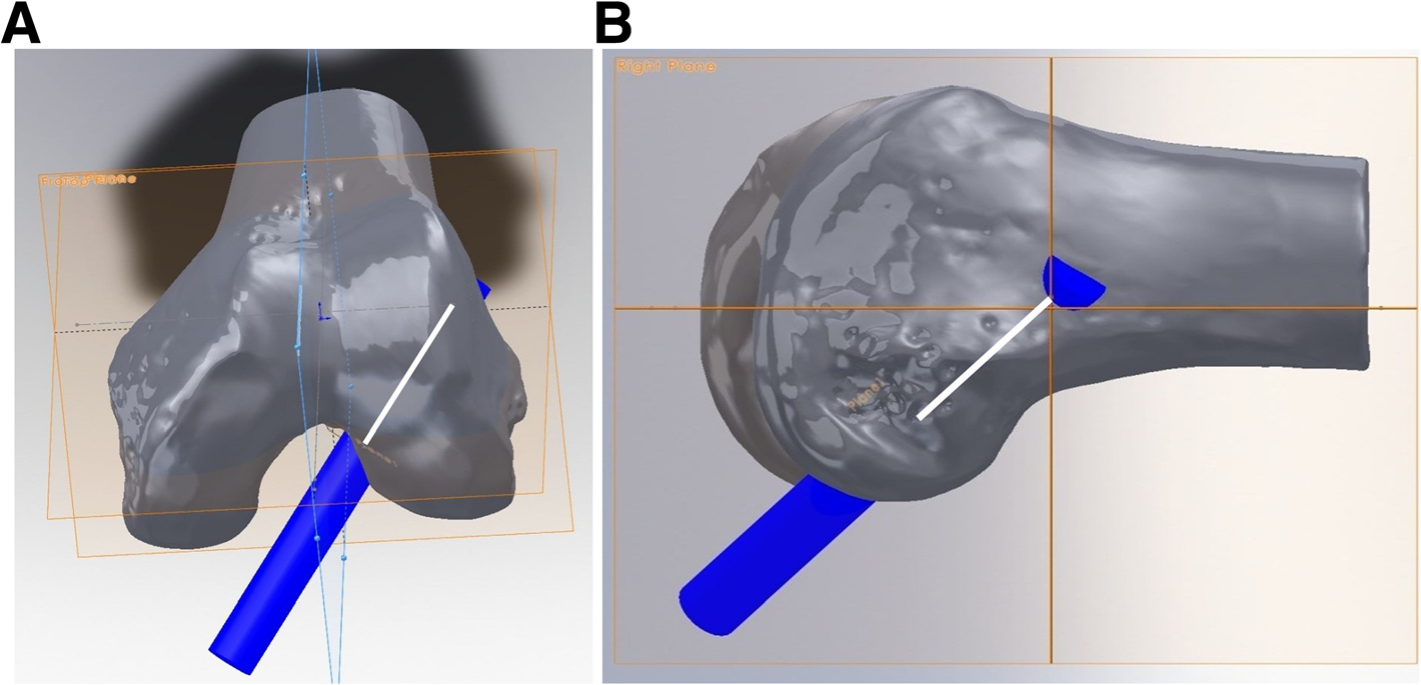
The length of the femoral tunnel measured without and with notchplasty

Notchplasty was created using SolidWorks® software (SolidWorks®, Corp., Waltham, MA, USA). There are various methods of performing notchplasty, but the authors considered a method of carrying up the notchplasty to the posterior cortex of the notch due to the broadly narrow notch from the anterior to the posterior. However, this type of notchplasty, which was actually performed, could not be implemented in practice because it was difficult to set the standard of bony resection through the 3D modeling program due to the uneven bony surface encountered. Since the authors were trying to confirm the effect of notchplasty on the femoral tunnel, the notchplasty was created with a depth of 3 mm and 5 mm at the ACL footprint based on the center of the ACL (Fig. 4). Finally, with the femoral tunnel through the center of the ACL femoral footprint, the effect of notchplasty (3 mm/5 mm) on the length and orientation of the femoral tunnel through the AM portal was measured (Fig. 5).

### Statistical analyses

A one-way analysis of variance (*α* = .05) was conducted to compare tunnel length according to notchplasty in each group. Multiple comparisons were conducted with a Tukey post hoc test. The anatomical center of the ACL footprint was compared between intact ACLs and notchplasty ACLs using an independent *t* test. An analysis of variance (ANOVA) test was also used to compare tunnel length without notchplasty, and 3 mm and 5 mm notchplasty. All statistical analyses were conducted using SPSS v.18.0 software (SPSS Inc., Chicago, IL, USA). Statistical significance was set at *P* < 0.05.

## Results

### The center of the ACL footprint

Based on Bernard’s quadrant method, without notchplasty, the center of the femoral footprint of the ACL was located at 33.85 ± 4.68% (range, 29.17 to 38.53) of a line parallel to Blumensaat’s line (*t*), and 35.84 ± 8.12% (range, 27.72 to 46.96) of a line perpendicular to Blumensaat’s line (*h*). After 3-mm notchplasty, the center changed to *t*: 34.09 ± 4.23% (range, 28.49 to 45.51)/*h*: 29.91 ± 9.83% (range, 14.04 to 42.58) [18]. In addition, the center changed to *t*: 34.21 ± 4.04% (range, 29.16 to 36.95)/*h*: 25.86 ± 10.36% (range, 8.64 to 38.77) after 5-mm notchplasty (Table 1). The center of the ACL footprint resulted in a significant change (*P* = 0.015), regardless of whether there was a 3-mm or 5-mm notchplasty. The results indicate movement of the high AM position to the coordinate axis, according to Bernard’s quadrant method.

**Table 1:** Anatomical center of the anterior cruciate ligament (ACL) footprint with and without notchplasty

	Before notchplasty		After 3-mm notchplasty		After 5-mm notchplasty		*P* value
Center of the ACL footprint (%)	*t*: 33.85 ± 4.68	*h*:35.84 ± 8.12	*t*:34.09 ± 4.23	*h*: 29.91 ± 9.83	*t*: 34.21 ± 4.04	*h*:25.86 ± 10.36	0.015

*t*: line parallel to Blumensaat’s line

*h*: line perpendicular to Blumensaat’s line

### Femoral-tunnel length with 3D drilling angle with and without notchplasty

In tunnels drilled at an axial angle of 70^o^ and a coronal angle of 20^o^, the mean femoral-tunnel length was 34.09 ± 2.99 mm (range, 29.8 to 37.7), 31.73 ± 2.47 mm (range, 27.3 to 35.8), and 30.38 ± 2.11 mm (range, 27 to 33.9), respectively (*P* = 0.002). The mean femoral-tunnel length when drilling at an axial angle of 60^o^ and a coronal angle of 30^o^ was 36.11 ± 2.28 mm (range, 32.4 to 40.5), 33.08 ± 2.15 mm (range, 28.9 to 36.6), and 31.26 ± 2.08 mm (range, 27.1 to 34.70), respectively (*P* < 0.001). At an axial angle of 45^o^ and a coronal angle of 45^o^, the mean femoral-tunnel length was 38.45 ± 3.63 mm (range, 30.6 to 42.2), 35.06 ± 3.25 mm (range, 28.8 to 42.5), and 32.98 ± 3.04 mm (range, 27.6 to 39.9), respectively (*P* < 0.001). The mean tunnel lengths of 70^o^ in the axial, 20^o^ in the coronal and 60^o^ in the axial, and 30^o^ in the coronal rotational axis groups were significantly shorter than that of the 45^o^, 45^o^ group (*P* < 0.001).

At an axial angle of 30^o^ and a coronal angle of 60^o^, the mean femoral-tunnel length was 46.28 ± 4.34 mm (range, 35.8 to 49.7), 42.78 ± 3.19 mm (range, 38.7 to 49.8), and 40.31 ± 3.36 mm (range, 35.9 to 48.1), respectively (*P* = 0.019). At an axial angle of 15^o^ and a coronal angle of 75^o^, the mean femoral-tunnel length was 54.01 ± 4.08 mm (range, 45.9 to 60), 52.87 ± 3.50 mm (range, 45.2 to 55.7) and 50.46 ± 3.13 mm (range, 44.3 to 54.1), respectively (*P* = 0.043). The mean tunnel lengths of both the 30^o^ in the axial, 60^o^ in the coronal and 15^o^ in the axial, and 75° in the coronal rotational axis groups were significantly greater than that of the 45^o^, 45^o^ group (*P* < 0.001) (Table 2).

**Table 2:** Femoral-tunnel length with 3-dimensional (3D) drilling angle through the anteromedial (AM) portal with and without notchplasty

3D entrance angle (coronal/axial/sagittal)	Before notchplasty (mm)	After 3 mm notchplasty (mm)	After 5 mm notchplasty (mm)	*P* value
20^o^/70^o^/45^o^	34.09 ± 2.99	31.73 ± 2.47	30.38 ± 2.11	0.002
30^o^/60^o^/45^o^	36.11 ± 2.28	33.08 ± 2.15	31.26 ± 2.08	< 0.001
45^o^/45^o^/45^o^	38.45 ± 3.63	35.06 ± 3.25	32.98 ± 3.04	< 0.001
60^o^/30^o^/45^o^	46.28 ± 4.34	42.78 ± 3.19	40.31 ± 3.36	0.019
75^o^/15^o^/45^o^	54.01 ± 4.08	52.87 ± 3.50	50.46 ± 3.13	0.043

## Discussion

The most remarkable finding of this study is that the femoral tunnel shortened with greater amounts of bone removal, a lower coronal plane angle, and a greater angle at the axial plane. Also, notchplasty can change the center of the ACL footprint. These results can be said to be clinically important considering that the placement of the femoral tunnel can affect the clinical outcome. Notchplasty is generally performed to improve visualization and to prevent impingement between the graft and bony structures when the notch is narrowed due to various causes [19–21]. Previously reported recommendations for notchplasty size have ranged from 2 mm to 25% of the lateral femoral condyle [22, 23]. However, recommendations related to the technical aspects of notchplasty are not well-described in the literature. How much bone to remove and whether to carry the notchplasty back to the posterior cortex of the notch are important concerns. For small osteophytes, relatively small amounts of bone removal will not be a problem. On the other hand, if notchplasty has to be carried to the posterior cortex of the notch due to a narrow notch, it may have a negative clinical effect. Notchplasty increases anterior tibial translation and decreases graft forces during low knee-flexion angles [24]. Another study also described notchplasty as having a greater effect on anterior stability than rotational stability, which could lead to failure of the ACLR [15]. This is because important bony landmarks for anatomical placement can be lost by notchplasty [16]. Markolf et al. compared the biomechanical effects of femoral notchplasty in ACLR with and without notchplasty [25]. They reported that after a notchplasty, a higher level of graft pre-tension will be necessary to restore normal laxity at 30o of flexion. However, there is, as yet, no anatomical study of the relationship between notchplasty and failed ACLR. Consequently, current methods of determining the position of the tunnel and technical aspects of the procedure, such as notchplasty, need to be challenged. The authors conducted this study on the assumption that bone removal to the posterior cortex of the notch was necessary due to the narrow notch. In this study, an alternative center of the ACL after 3-mm and 5-mm deep, notchplasty was placed using the original center of the ACL by computer simulation. Furthermore, this study shows a shortened femoral-tunnel length with greater notchplasty deepening before standard notchplasty, 3-mm notchplasty and 5-mm notchplasty. Especially, the femoral-tunnel length was almost 30 mm at the low coronal angle and the high axial angle. Among the five models, the 3D angle plane with an extreme range, such as 20^o^ in the coronal, 70^o^ in the axial, and 45^o^ in the transverse, more significantly influenced the result of the 5-mm notchplasty. This can eventually reduce the graft strength, so it would seem to have clinical significance.

Contemporary studies have recommended drilling the femoral tunnel at the anatomical ACL origin to enhance rotatory stability of the reconstruction [26]. The disadvantage of the TT technique is that a vertical tunnel is formed, and rotatory instability remains, but using the AM portal technique complements this rotatory instability, but forms an oblique, horizontal, short femoral tunnel [7, 27]. Increasing femoral-tunnel obliquity results in a short femoral tunnel and increases the risk of posterior tunnel-wall blowout [6, 28–30]. Also recent papers report complications of protrusions of inserted fixers [7, 31, 32]. The trend towards anatomical ACLR has resulted in a shorter femoral tunnel, which may have implications regarding the strength of the reconstruction. Greis et al. showed that both tendon fit and tendon length in the femoral tunnel were proportionate to pullout strength in a dog model at 6 weeks after reconstruction [33]. The minimal length necessary within the femoral tunnel has not been substantiated in human models but increasing femoral-tunnel length may increase the strength of the reconstruction and maximizes the fixation options to accommodate the shorter femoral tunnels that should be developed. Therefore, when anatomical ACLR is performed, it is very important to make the shortened femoral tunnel as long as possible. Our study shows that femoral-tunnel length can be optimized by altering the axial drilling angle and coronal angles. Our results suggest that when drilling the femoral tunnel through the AM approach, an axial angle of 45^o^, 60^o^, and 70^o^ and a coronal angle of 20^o^, 30^o^, and 45^o^ results in a significantly shorter femoral tunnel when compared with tunnels drilled at an axial angle of 15^o^ and 30^o^ and a coronal angle of 60^o^ and 75^o^. However, the tunnel was too vertical at a 60^o^ and 75^o^ coronal angle while the length was secured to around 45–55 mm. But, these vertical angles would not be recommended as rotational instability may remain. Therefore, considering the case of the coronal angles of 20^o^, 30^o^, and 45^o^, as the angle increases, the femoral-tunnel length becomes longer, so a smaller coronal angle should be avoided.

Although there are many biomechnical and anatomical studies that study the effects of femoral tunnel length, femoral tunnel orientation, and internal fixation devices in ACLR, this study is the first study to confirm the change of femoral tunnel length and orientation according to notchplasty, by using an accessory AM portal with a 3D drilling angle. This kind of study is rare because the center of the ACL is hard to detect under the situation of anatomical ACLR with notchplasty, and the wrong positioning of the guide pin can alter the direction of the femoral tunnel. Fu et al. also suggested that a large lateral notchplasty may displace the femoral insertion of the ACL too laterally, leading to abnormal knee kinematics and poor functional outcomes [34]. Seo et al. suggested that an enlarged tunnel orifice may lead to a discrepancy between the tunnel and the graft at the tunnel aperture [35]. Also, the bone removal of the inner position and roof of the lateral femoral condyle during notchplasty can induce a more shortened femoral-tunnel length [36, 37]. In this study, notchplasty can change the center of the ACL footprint. This change was mainly noticeable in height. In particular, the greater the depth of the notchplasty, the greater this change. This eventually leads to non-anatomical ACLR, which has the potential to be detrimental to the clinical results.

The present study has several limitations that require consideration. First, this study was conducted in a small sample size due to the nature of the anatomical study, and the cadavers used in our study are relatively old, which generally has limitations that cannot represent the generally young age of ACLR patients. The second weakness is that the anatomical study is a time-zero study, and the effect of ACL on the biological response to ACLR is not known. Third, the study underwent virtual computer simulation to measure the length of the femoral tunnel by using a relatively narrow range of compounded 3D drilling angles. This is a limitation since our arranged angles contain the angle that can be used in the real procedure due to impingement between the drill bit and the medial femoral condyle or meniscus. However, we think that this angle setting may eventually lead to a better understanding of the results of this study. The importance of this study is that the change of the femoral tunnel due to notchplasty and 3D drilling angle can be confirmed using a computer program that can be accurately evaluated through direct visualization of the length of the femoral tunnel without extensive dissection of cadaveric soft tissues.

## Conclusions

Excessive notchplasty can lead to a non-anatomical center of the ACL footprint, which results in non-anatomical ACLR, and requires prudence because it shortens the femoral-tunnel length. Three-dimensional drilling of the femoral tunnel through the AM portal at extremely high or low coronal and axial angles with notchplasty produces an inappropriate tunnel length. Therefore, if notchplasty is necessary when performing anatomical ACLR, bone removal should be minimized and a standard femoral angle should be obtained.

## Data Availability

Not applicable

## Abbreviations

ACL: Anterior cruciate ligament
ACLR: Anterior cruciate ligament reconstruction
CT: Computer tomography
TT: Transtibial
AM: Anteromedial
